# Insulin Resistance Is Not Associated with an Impaired Mitochondrial Function in Contracting Gastrocnemius Muscle of Goto-Kakizaki Diabetic Rats *In Vivo*

**DOI:** 10.1371/journal.pone.0129579

**Published:** 2015-06-09

**Authors:** Michael Macia, Emilie Pecchi, Christophe Vilmen, Martine Desrois, Carole Lan, Bernard Portha, Monique Bernard, David Bendahan, Benoît Giannesini

**Affiliations:** 1 Aix-Marseille Université, CNRS, CRMBM UMR 7339, 13385, Marseille, France; 2 Universitx Paris-Diderot, Sorbonne Paris Cité, Laboratoire B2PE, Unité BFA, CNRS EAC 4413, Paris, France; Universidad Pablo de Olavide, Centro Andaluz de Biología del Desarrollo-CSIC, SPAIN

## Abstract

Insulin resistance, altered lipid metabolism and mitochondrial dysfunction in skeletal muscle would play a major role in type 2 diabetes mellitus (T2DM) development, but the causal relationships between these events remain conflicting. To clarify this issue, gastrocnemius muscle function and energetics were investigated throughout a multidisciplinary approach combining *in vivo* and *in vitro* measurements in Goto-Kakizaki (GK) rats, a non-obese T2DM model developing peripheral insulin resistant without abnormal level of plasma non-esterified fatty acids (NEFA). Wistar rats were used as controls. Mechanical performance and energy metabolism were assessed strictly non-invasively using magnetic resonance (MR) imaging and 31-phosphorus MR spectroscopy (^31^P-MRS). Compared with control group, plasma insulin and glucose were respectively lower and higher in GK rats, but plasma NEFA level was normal. In resting GK muscle, phosphocreatine content was reduced whereas glucose content and intracellular pH were both higher. However, there were not differences between both groups for basal oxidative ATP synthesis rate, citrate synthase activity, and intramyocellular contents for lipids, glycogen, ATP and ADP (an important *in vivo* mitochondrial regulator). During a standardized fatiguing protocol (6 min of maximal repeated isometric contractions electrically induced at a frequency of 1.7 Hz), mechanical performance and glycolytic ATP production rate were reduced in diabetic animals whereas oxidative ATP production rate, maximal mitochondrial capacity and ATP cost of contraction were not changed. These findings provide *in vivo* evidence that insulin resistance is not caused by an impairment of mitochondrial function in this diabetic model.

## Introduction

Type 2 diabetes mellitus (T2DM) is a metabolic disorder characterized by chronic hyperglycemia leading to long-term damage, dysfunction and failure of various organs, especially pancreas, heart, skeletal muscle and blood vessels. T2DM is initially caused by peripheral insulin resistance syndrome, i.e., the inability of insulin to stimulate glucose absorption in peripheral tissue, in association with the progressive failure of the pancreatic cells to supply a sufficient amount of insulin [1]. The development of insulin resistance is itself caused by marked disturbances in insulin signaling induced by excess intake of carbohydrates and fats [2].

It is noteworthy that skeletal muscle plays a predominant role in the development of insulin resistance because it is one of the major organs participating in the assimilation, storage and utilization of glucose provided by food [3]. Insulin resistance causes in return a reduction of both exercise tolerance and mechanical performance [4]. Lipid metabolism alteration and mitochondrial dysfunction in skeletal muscle have been implicated in the etiology of T2DM but the causal relationships with insulin resistance development are still unclear [5–8]. Lipid oxidation has been reported to be increased in the early stage of insulin resistant state [9] while increased non-esterified fatty acids (NEFA) plasma level would inhibit skeletal muscle glucose uptake and glycogen synthesis [10]. Further, it has been clearly disclosed that the increase in intramyocellular lipids (IMCL) content reduces insulin sensitivity [11–13]. Besides, a link between mitochondrial function impairment and insulin resistance development is highly suspected [7]. On the basis of biochemical [14–16], gene expression [17, 18] and *in vivo* 31-phosphorus magnetic resonance spectroscopy (^31^P-MRS) [7, 8, 19, 20] measurements, it has been initially proposed that mitochondrial capacity reduction contributes to IMCL accumulation thereby leading to insulin signaling failure and insulin resistance development. For instance, Bonnard et al. [21] compared mitochondrial density in mice receiving a high-fat-high-sucrose diet (HFHSD) and KKAγ mice, a genetic model of obesity and diabetes with a normal plasmatic NEFA. Interestingly, mitochondrial density and structure were abnormal only in the HFHSD model but not in the KKAγ strain, thereby suggesting a direct link between the increased plasmatic NEFA level and the mitochondrial number and integrity [21].

On the contrary, a growing number of studies has proposed that mitochondrial impairment is not the causative factor of insulin resistance development [22–27]. In a recent review, Holloszy [25] underlined that mitochondrial biogenesis increases in rodents receiving a high-fat diet, and mitochondrial deficiency severe enough to impair fat oxidation in resting muscle causes an increase, not a decrease, in insulin action. Overall, the causal link between mitochondrial function and lipid metabolism in the context of T2DM is still a matter of debate. However, it is noteworthy that most of the studies mentioned above have investigated mitochondrial density and enzymes activity but not mitochondrial function *per se*. Under these conditions, energy requirement is minimal and regulatory mechanisms of energy production have not been investigated. Data regarding *in vivo* mitochondrial function under conditions of high-energy demand such as in exercising muscle are then missing.

The purpose of the present study was to investigate mitochondrial function in electrically stimulated muscle of diabetic GK rat, a non-obese T2DM model displaying insulin resistance and normal NEFA plasmatic level [28–32]. Metabolic fluxes were measured strictly non-invasively with respect to mechanical performance using magnetic resonance (MR) imaging and ^31^P-MRS, and mitochondrial content was evaluated on the basis of citrate synthase activity. The corresponding results were analyzed together with measurement of plasmatic levels of insulin, glucose and NEFA, IMCL and intramuscular contents for ATP, glycogen and glucose.

## Materials and Methods

### Animal care and feeding

Wistar (WT; *n* = 8) and Goto-Kakizaki [GK/Par subline [33]] (*n* = 10) 7-month-old male rats were used for these experiments. All animal work and care were carried out in strict accordance with the guidelines of the European Communities Council Directive 86/609/EEC for Care and Use of Laboratory Animals with the approval of the animal experiment committee of Aix-Marseille University. Animals were socially housed as 2–3 per cage in an environmentally controlled facility (12–12 h light-dark cycle, 22°C) with free access to commercial standard food (diet 113; SAFE, Augy, France) and water until the time of the experiment. Every attempt was made to minimize the number and the suffering of animals.

### *In vivo* investigation of gastrocnemius muscle function and energetics

#### Animal preparatio

Rats were initially anesthetized in an induction chamber with 4% isoflurane (Forene; Abbott France, Rungis, France) mixed in 33% O_2_ (0.5 L/min) and 66% N_2_O (1 L/min). The right lower hindlimb was shaved and electrode cream was applied at the knee and heel levels in order to optimize transcutaneous electrical stimulation. Anesthetized animal was placed supine in a home-built cradle allowing the strictly non-invasive MR investigation of gastrocnemius muscle function and energetics [34]. Briefly, the setup integrates an ergometer consisting of a foot pedal coupled to a force transducer, and two rod-shaped transcutaneous surface electrodes (located above the knee and at the heel level, respectively) connected to an electrical stimulator (Type 215/T; Harvard Apparatus, Germany). Corneas were protected from drying by applying ophthalmic cream and animal’s head was placed in a facemask continuously supplied with 2.5% isoflurane in 33% O_2_ (0.2 L/min) and 66% N_2_O (0.4 L/min) throughout the experiment. The facemask was connected to an open-circuit gas anesthesia machine (Isotec 3, Ohmeda Medical, Herts, UK). Exhaled and excess gases were removed through a canister filled with activated charcoal (Smiths Industries Medical System, London, UK) mounted on an electrical pump extractor (Equipement Vétérinaire Minerve, France). The hindlimb was centered inside a 30 mm-diameter ^1^H-MR Helmholtz imaging coil so that the belly of the gastrocnemius muscle located above an elliptic (10 x 16 mm) ^31^P-MRS surface coil and the foot was positioned on the pedal of the ergometer. The gastrocnemius muscle was passively stretched at rest by adjusting the pedal position, thereby modifying the angle between the foot and the lower hindlimb in order to produce a maximum isometric twitch tension in response to a supramaximal square wave pulses (1 ms duration; 6–8 mA). During experiment, animal body temperature was controlled and maintained within a physiological range during anesthesia, using a feedback loop including an electrical heating blanket (Prang+Partner AG, Pfungen, Switzerland), a temperature control unit (Ref. No. 507137, Harvard Apparatus, Les Ulis, France) and a rectal probe (Ref. No. 507145, Harvard Apparatus, Les Ulis, France).

#### Muscle electrostimulation protocol and force output measurement

Mechanical performance was assessed during a standardized fatiguing protocol consisting in 6 min of repeated maximal isometric contractions induced electrically with square-wave pulses (6–8 mA, 1 ms duration) at a frequency of 3.3 Hz. Electrical signal coming out from the ergometer was amplified with a home-built amplifier (Operational amplifier AD620; Analog Devices, Norwood, MA, USA; gain = 70 dB; bandwidth 0–5 kHz) and converted to a digital signal (PCI-6220; National Instrument, Austin, TX, USA) that was continuously monitored and recorded on a personal computer using the WinATS 6.5 software (Sysma, Aix-en-Provence, France). Absolute isometric force production was calculated by integrating the isometric tension (in N) with respect to time, and was expressed as tension-time integral (in N*s). Specific force was defined as the absolute force normalized by muscle volume calculated from hindlimb MR images.

#### MR data acquisition

MR explorations were done in the 4.7-Tesla horizontal magnet of a 47/30 Biospec Avance MR system (Bruker, Karlsruhe, Germany). Sixteen consecutive non-contiguous axial images (1 mm thickness, spaced 0.5 mm) were acquired across the resting lower hindlimb using a rapid acquisition relaxation-enhanced (RARE) sequence (8 echoes; effective echo time = 49.3 ms; actual echo time = 16 ms; repetition time = 2000 ms; 30 x 32 mm field of view; 256 x 192 matrix size). ^31^P-MR spectra (16 accumulations; 1.8 s repetition time; 8 kHz spectral width, 512 data points) from the gastrocnemius muscle region were continuously acquired during 6 min of rest, 6 min of electrostimulation (ES) and 16 min of post-ES recovery. MR data acquisition was gated to muscle ES in order to reduce potential motion artifacts due to contraction. A fully relaxed spectrum (12 scans, 20 s repetition time) was acquired at rest, followed by a total of 768 saturated free induction decays (FID) (1.875 s repetition time). The first 64 FIDs were acquired at rest and summed together. The next 192 FIDs were acquired during the ES period and were summed by packets of 32, allowing a temporal resolution of ∼60 s. The remaining 512 FIDs were obtained during the post-ES recovery period and were summed as 7 packets of 32 FIDs followed by 3 packets of 64 FIDs and one packet of 96 FIDs.

#### MR data processing

**Fig 1:**
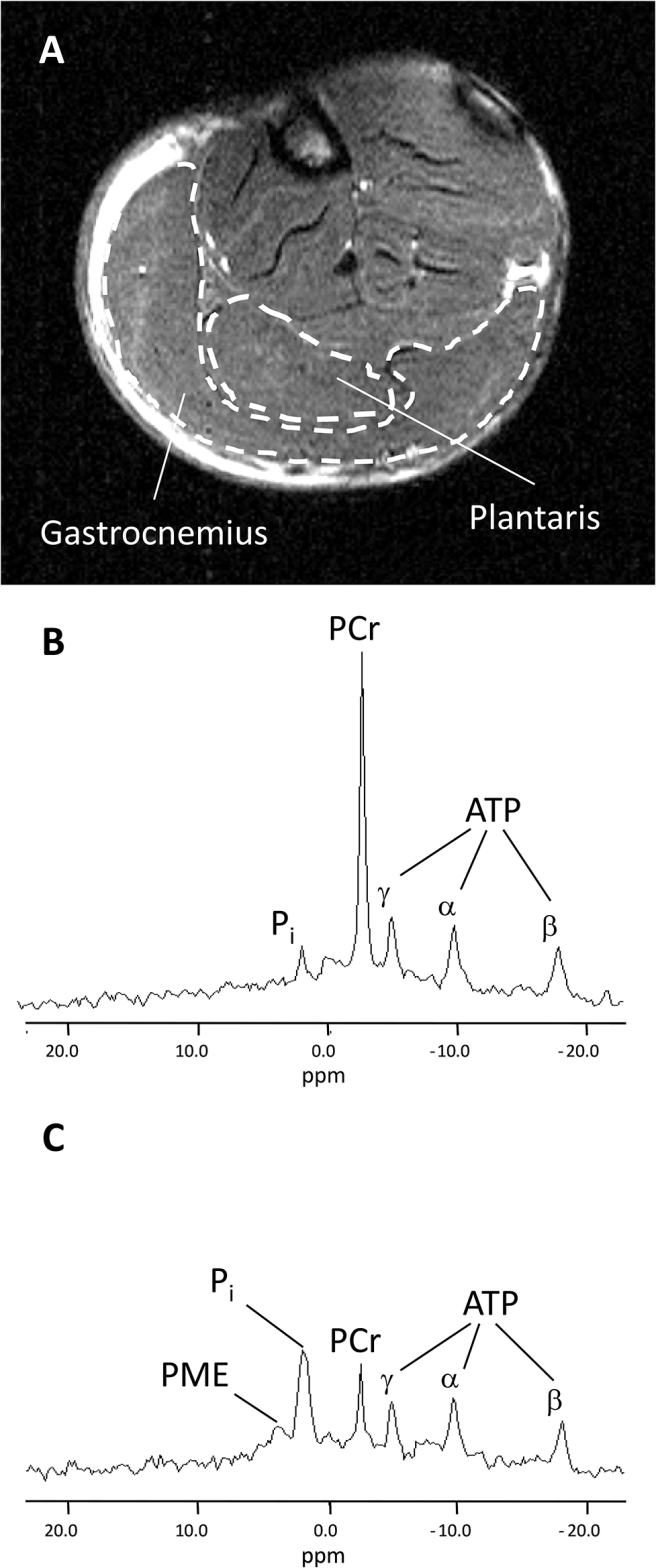
Typical transversal slice obtained by MRI of a rat lower hindlimb (A), ^31^P-MRS spectra obtained from a single WT rat gastrocnemius muscle at rest (B) and at the end of 6-min of *in vivo* electrostimulation protocol (C). Abbreviations for signal assignments (in p.p.m.) are PME (phosphomonoester), P_i_ (inorganic phosphate), PCr (phosphocreatine), and γ-, α-, and β-resonances of ATP.

MR data were processed using a custom-written image analysis program developed with the IDL software (Interactive Data Language, Research System, Inc., Boulder, CO, USA). For each MR image, regions of interest were manually outlined using the DISPIMAG software [35] so that the corresponding cross-sectional areas could be measured (Fig 1A). Gastrocnemius muscle volume was then calculated as the sum of the volumes included between the consecutive slices. Relative concentrations of phosphocreatine (PCr), inorganic phosphate (P_i_) and β-ATP were obtained by a time-domain fitting routine using the AMARES-MRUI Fortran code [36] and appropriate prior knowledge of the ATP multiplets. Signal areas were corrected for magnetic saturation using fully relaxed spectra collected at rest with a repetition time of 20 s. Absolute concentrations of phosphorylated compounds were expressed relative to a resting β-ATP concentration determined by bioluminescence as detailed below. Intracellular pH (pH_i_) was calculated from the chemical shift of the P_i_ relative to the PCr signal [37]. Time-points for the time course of phosphorylated metabolite concentrations and pH_i_ were assigned to the midpoint of the acquisition interval.

#### Metabolic fluxes calculations

ATP productions from the different pathways (PCr degradation via CK reaction, mitochondrial oxidative phosphorylation and glycolysis) were calculated as described previously [38–40]. ATP cost of contraction was calculated as the rate of ATP production scaled to force output during the same period of time.

ATP production rate from PCr degradation via creatine kinase (CK) reaction (*D*) was directly calculated using the [PCr] time-course throughout the stimulation period: *D* = -dPCr/d*t*.

Oxidative ATP production rate (*Q*) was calculated considering that ADP stimulates oxidative ATP synthesis throughout a hyperbolic relationship: *Q* = *Q*_max_/(1+*K*_*m*_/[ADP]), in which *K*_*m*_ (the ADP concentration at half-maximal oxidation rate) is 50 μM as previously reported in rat gastrocnemius muscle [41] and *Q*_max_ is the maximal oxidative capacity. [ADP] was calculated from [PCr], [ATP] and pH_i_ using the CK equilibrium constant (*K* = 1.67 10^9^ M^-1^) [42]. *Q*_max_ was calculated using the rate of PCr resynthesis at the start of the post-stimulation recovery period (*V*PCr_rec_) and the concentration of free cytosolic ADP measured at the end of the stimulation period: *Q*_max_ = *V*PCr_rec_ (1 + *K*_*m*_/[ADP]_end_). *V*PCr_rec_ was the product of *k* (the pseudo-first-order rate-constant of PCr recovery) and [PCr]_cons_ (the amount of PCr consumed at the end of the stimulation period). In order to determine *k*, the PCr time-course during the post-stimulation recovery period was fitted to a first-order exponential curve: [PCr]_t_ = [PCr]_rest_—[PCr]_cons_ e^-*k*t^, where [PCr]_rest_ is the concentration of PCr measured at rest.

Glycolytic ATP production rate (*L*) was inferred considering that, when coupled to ATP hydrolysis, glycolytic ATP production is related to proton production (*H*_Gly_) with a stoichiometry of 1.5 moles ATP per proton (*L* = 1.5 *H*_Gly_) [43]. This proton production can be calculated from the observed changes in pH_i_ and taking into account protons consumed by PCr degradation throughout the CK reaction (*H*_CK_), passively buffered in the cytosol (*H*_β_), leaving the cell (rate of net proton efflux, *H*_Efflux_) and produced by oxidative phosphorylation (*H*_Ox_): *H*_Gly_ = *H*_CK_ + *H*_β_ + *H*_Efflux_—*H*_Ox_. Calculation of *H*_CK_ was done from the time-dependent changes in [PCr] and with the stoichiometric coefficient *φ* = 1/(1+10^(pHi-6.75)^), which represents the number of protons associated to P_i_ production [44]: *H*_CK_ = *φ* dPCr/d*t*. Besides, *H*_β_ was the product of *β*_total_ (in Slykes, millimoles acid added per unit change in pH_i_) and pH_i_ changes (ΔpH_i_ = pH_observed_—pH_rest_): *H*_β_ = (-*β*_total_ΔpH_i_). The apparent buffering capacity (*β*_total_) takes into account the buffering capacity of P_i_ (*β*_Pi_) and the buffering capacity of muscle tissue (*β*_tissue_): *β*_total_ = *β*_Pi_ +*β*_tissue_, where *β*_Pi_ = 2.3[P_i_]/((1+10^(pHi-6.75)^)(1+10^(6.75-pHi)^)) [44]. It has been shown that *β*_tissue_ varies linearly between pH 7 (16 Slykes) and pH 6 (37 Slykes) in murine gastrocnemius muscle [45]. Accordingly, *β*_tissue_ was calculated as follows: *β*_tissue_ = -21pH_i_+163. During muscle stimulation, *H*_efflux_ was calculated using the proportionality constant *λ* (in μmol/s/pH unit) referring to the ratio between the rate of proton efflux and pH_i_: *H*_efflux_ = -*λ*ΔpH_i_. This constant was determined at the start of the post-stimulation recovery period as *λ* = -*V*_eff_ /ΔpH_i_. At that time, although protons are generated throughout the aerobic PCr resynthesis, pH_i_ recovers back to basal because of net proton efflux from the cell: *H*_efflux_ can then be calculated taking into account proton loads associated with CK reaction and mitochondrial ATP synthesis on the one hand and the rate of pH changes on the other hand. *H*_efflux_ = *H*_CK_ + *H*_Ox_ +*β*_total_dpH_i_/d*t*. The rate of aerobic proton production coupled to oxidative ATP synthesis was quantified as follows [44]: *H*_Ox_ = *mV*PCr_rec_, with *m* = 0.16/(1+10^(6.1-pH)^).

### *In vitro* analytical procedures

After MR experiments, transcardiac blood samples (0.25 ml) were obtained with a thin needle carefully introduced into the heart during the anesthetic epoch. Plasma was immediately separated after blood centrifugation (15 min at 4,000 rpm) in EDTA-treated tubes. Afterwards, anesthetized animals were immediately euthanized by cervical dislocation, and gastrocnemius muscles were quickly removed, freeze-clamped with liquid nitrogen-chilled metal tongs and stored at -80°C.

Plasmatic concentrations of insulin, glucose and NEFA were measured using insulin (Mercodia, Uppsala, Sweden), glucose (Randox Laboratories, Crumlin, Antrim, UK) and NEFA (Roche Diagnostics, Roche Applied Science, Mannheim, Germany) determination kits.

Intramuscular contents for ATP, glycogen and glucose were determined in freeze-clamped gastrocnemius muscles (50 to 60 mg) homogenized in ice-cold 0.6 M perchloric acid (1.2 ml) with a Polytron PT2100 (Kinematica AG, Luzern, Switzerland). After incubation for 15 min at 4°C, the homogenate was centrifuged (15 min, 2000 x *g*, 4°C) and the supernatant was neutralized with K_2_CO_3_ and placed for 30 min at 4°C. ATP concentration was measured using a bioluminescence-based determination kit (ref. A22066, Invitrogen, Eugene, Oregon, USA), and glycogen and glucose contents were assessed by colorimetric procedure (ref. E2GN-100, EnzyChrome, Hayward, California, USA).

IMCL content was determined in freeze-clamped gastrocnemius muscle (50 to 70 mg) homogenized in 1 ml of a 1% (w/v) Triton X-100 in chloroform solution. Briefly, homogenate was centrifuged (10 min, 13000 x *g*, 20°C), the organic phase was collected and the chloroform was removed using a nitrogen evaporator (N-EVAP-111, Organomation, Berlin, Massachusetts, USA). IMCL content was then measured using a colorimetric detection kit (ref. MAK044, Sigma-Aldrich, St. Louis, Missouri, USA).

Citrate synthase activity was measured (ref. CS0720, Sigma-Aldrich, St. Louis, Missouri, USA) in another part (20 to 30 mg) of the freeze-clamped muscle, which was homogenized with a lysis reagent (ref. C3228, Sigma-Aldrich, St. Louis, Missouri, USA) and a protease inhibitor cocktail (P8340, Sigma-Aldrich, St. Louis, Missouri, USA).

### Statistical analysis

Data were analyzed with JMP software (SAS Institute Inc., Cary, North Carolina, USA). For variables changing with respect to time during ES and post-ES recovery periods, overall time-courses were analyzed with two-way (group x time) analyses of variance (ANOVAs) with repeated measures on time. When appropriate, post-hoc Tukey tests were used in order to identify differences at each time-point. Other variables were compared using unpaired two-tailed Student's t-test. *P* < 0.05 was considered as significant. Values are presented as means ± SE.

## Results

### Animal characteristics

The corresponding data are summarized in Table 1. Body weight and gastrocnemius muscle volume were lower (-14% and -15%, respectively) in GK rats, but body weight-to-gastrocnemius volume ratio was similar between both groups. Plasmatic and muscle glucose contents were higher (+79% and +23%, respectively) in GK animals whereas plasma insulin was lower (-37%). On the other hand, there were no differences between both groups for NEFA, IMCL and intramuscular glycogen contents.

**Table 1:** Animal characteristics.

	WT	GK	*P*-value
Body weight (g)	491 ± 16	423 ± 4	< 0.001
Gastrocnemius muscle volume (cm^3^)	1.84 ± 0.04	1.56 ± 0.02	< 0.001
BW/GV (g/cm^3^)	268 ± 10	272 ± 3	n.s.
*Plasma*			
Insulin (ng/ml)	3.4 ± 0.3	2.2 ± 0.2	< 0.01
Glucose (mM)	10.9 ± 0.6	19.4 ± 1.9	< 0.001
NEFA (mM)	0.22 ± 0.02	0.21 ± 0.02	n.s
*Gastrocnemius muscle*			
Glycogen (μmol/g)	20.5 ± 1.4	20.2 ± 1.5	n.s.
Glucose (μmol/g)	8.1 ± 0.4	10.0 ± 0.7	0.031
IMCL (μmol/g)	1.8 ± 0.1	1.6 ± 0.1	n.s.

Data are means ± SEM; n.s., not statistically significant; BW/GV, body weight/gastrocnemius muscle volume ratio.

### Mechanical performance

**Fig 2:**
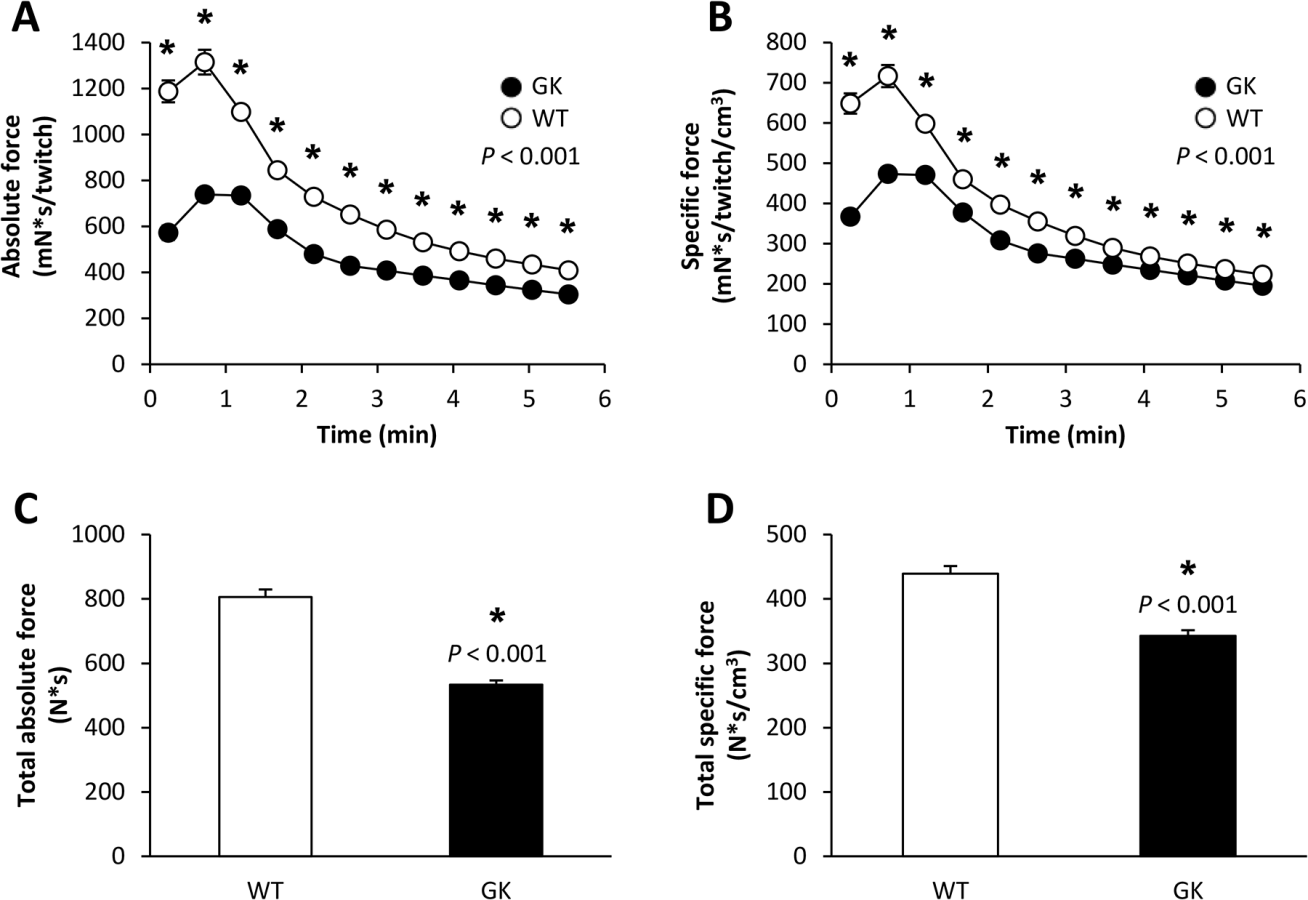
Time-dependent changes in absolute (A) and specific (B) force during the 6-min *in vivo* electrostimulation protocol, total amount of absolute (C) and specific (D) force produced during the whole protocol. Values are means ± SEM. ^*^Significant difference between GK and WT.

For GK and WT animals, both absolute and specific force displayed a biphasic profile with an initial transient increase in the early stage of the 6-min ES period followed by a progressive decrease until the end of the ES protocol as a sign of fatigue development (Fig 2A and 2B). At this stage, the extent of force reduction was significantly lower (-14%) in GK animals. Overall, the total absolute and specific force developed during the whole 6-min ES period were both lower (-34% and -22%, respectively) in the GK group (Fig 2C and 2D).

### Metabolic changes

Typical ^31^P-MR spectra recorded from a single WT gastrocnemius muscle are presented in Fig 1B and 1C. At rest, [PCr] and PCr/ATP ratio were lower (-23% and -21%, respectively) in the GK group whereas pH_i_ was higher (Table 2). On the other hand, there were no differences between both groups for [ATP], [ADP] and citrate synthase activity (Table 2).

**Table 2:** Energy metabolism of gastrocnemius muscle.

	WT	GK	*P*-value
*Rest*			
[PCr]/[ATP]	4.4 ± 0.2	3.5 ± 0.2	0.005
PCr (μmol/g)	32 ± 2	24 ± 1	0.002
ATP (μmol/g)	7.22 ± 0.04	7.06 ± 0.4	n.s.
ADP (nmol/g)	8.8 ± 0.2	10.4 ± 0.7	n.s.
pH_i_	7.06 ± 0.01	7.14 ± 0.03	0.026
Citrate synthase activity (mmol/g/min)	0.27 ± 0.04	0.24 ± 0.03	n.s.
*Onset of the stimulation period*			
*V*PCr_stim_ (μmol/g/min)	63 ± 4	37 ± 3	< 0.001
*k*PCr_stim_ (min^-1^)	2.4 ± 0.1	2.2 ± 0.2	n.s.
*End of the stimulation period*			
PCr (μmol/g)	7 ± 1	10 ± 1	0.031
ΔPCr_cons_ (%)	78 ± 1	58 ± 4	< 0.001
ADP (nmol/g)	23 ± 1	21 ± 2	n.s.
pH_i_	6.34 ± 0.03	6.41 ± 0.03	n.s.
ΔpH_i_ (pH units)	0.72 ± 0.03	0.73 ± 0.04	n.s.
*Recovery*			
*V*PCr_rec_ (μmol/g/min)	8 ± 1	6 ± 1	n.s.
*k*PCr_rec_ (min^-1^)	0.38 ± 0.04	0.39 ± 0.04	n.s.
*Q*_max_ (μmol/g/min)	26 ± 4	22 ± 3	n.s.

Data are means ± SEM; n.s., not statistically significant; *V*PCr_stim_, initial rate of PCr breakdown at the start of the 6-min stimulation period; *k*PCr_stim_, PCr breakdown rate constant at the start of the 6-min stimulation period; ΔPCr_cons_, PCr consumption at the end of 6-min stimulation period; *V*PCr_rec_, initial rate of PCr resynthesis at the start of the post-stimulation period; *k*PCr_rec_, PCr resynthesis rate constant at recovery period start; *Q*_max_, maximal oxidative capacity.

**Fig 3:**
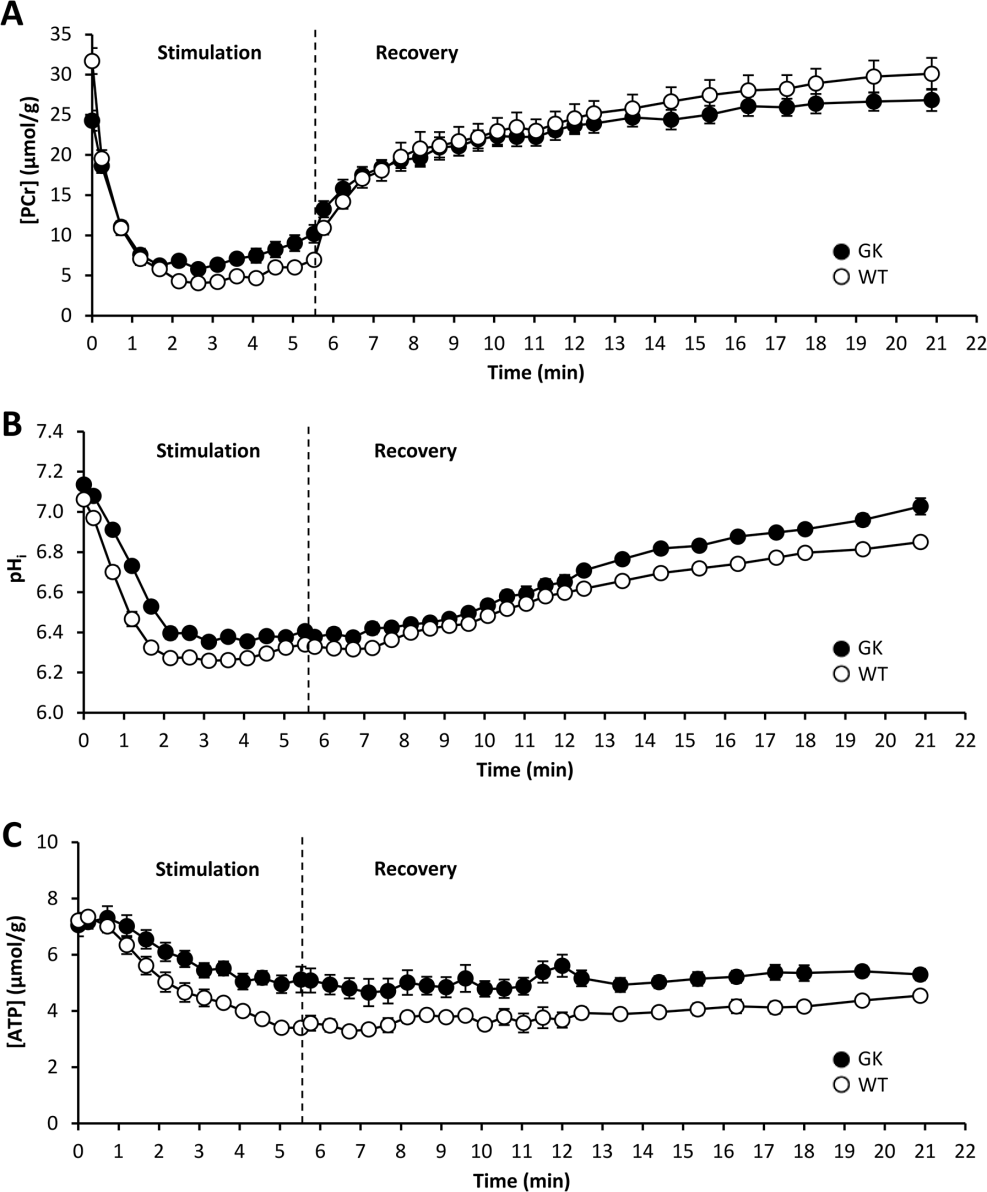
Time-dependent changes in gastrocnemius muscle [PCr] (A), intracellular pH (B) and [ATP] (C) during 6 min of *in vivo* electrostimulation and 15 min of post-electrostimulation recovery periods. The first point (t = 0) indicates the measured value during the resting period. Values are means ± SEM.

At the onset of the ES protocol, PCr was rapidly consumed (Fig 3A) at an initial rate that was significantly lower in the GK group (Table 2). Afterward, PCr level reached a steady state that was maintained until the end of the ES period. At this time, the extent of PCr consumption (ΔPCr) was 25% lower in the GK group (Table 2). During the first 3 minutes of the ES period, pH_i_ fell rapidly to reach a steady state that was fairly constant during the remaining ES period (Fig 3B). The extent of acidosis (ΔpH_i_) at the end of the ES period was similar between both groups (Table 2). For each group, ATP level decreased linearly throughout the ES protocol, but to a lower extent in GK animals (P < 0.005, Fig 3C).

During the post-ES recovery period, phosphorylated compounds and pH_i_ progressively reached their respective basal values (Fig 3). The maximal oxidative capacity (*Q*_max_) and both the initial rate (*V*PCr_rec_) and rate constant (*k*PCr_rec_) of PCr resynthesis were similar in GK and control groups (Table 2).

### Metabolic fluxes and ATP cost of contraction

**Fig 4:**
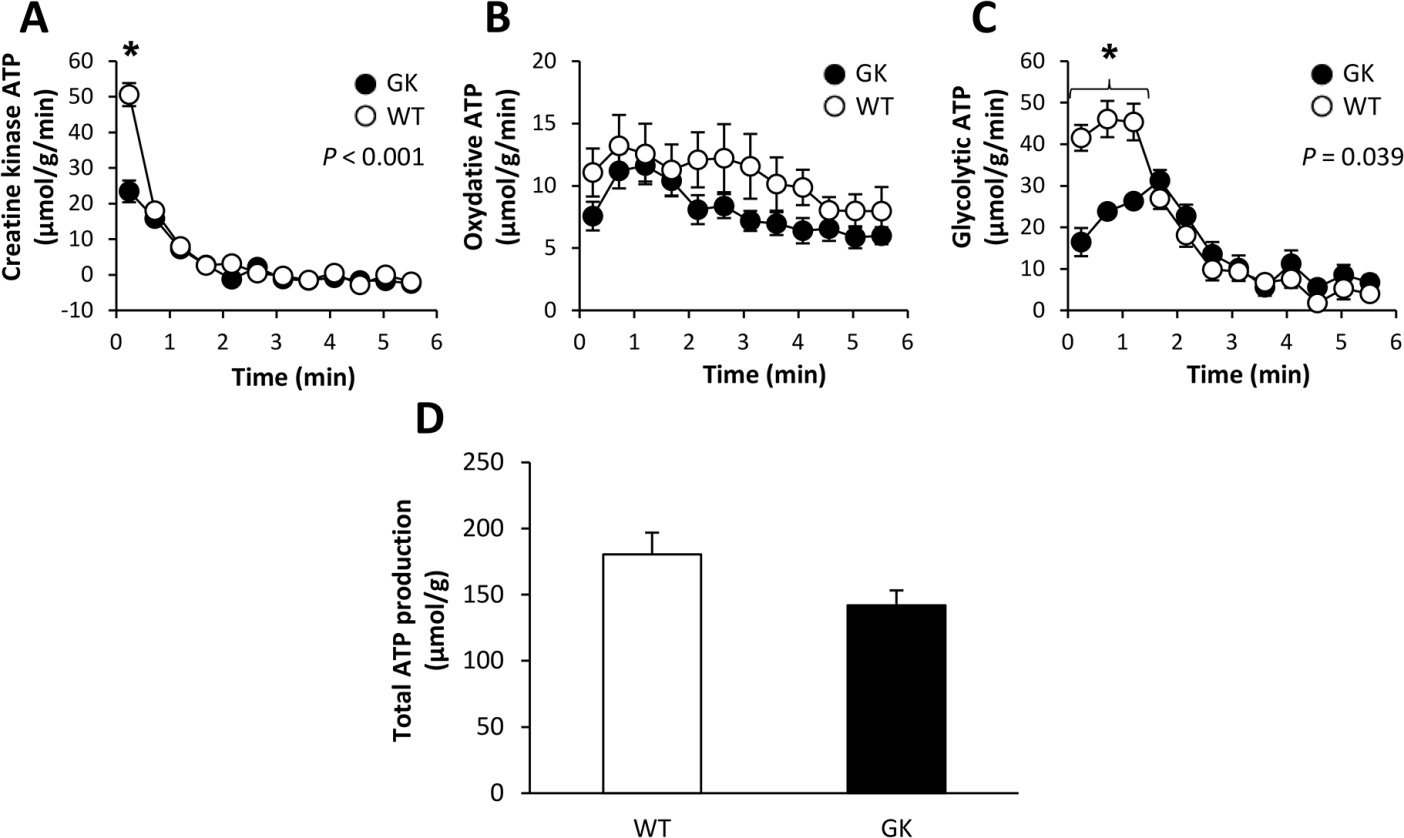
Rates of ATP production from CK reaction (A), oxidative phosphorylation (B) and glycolysis (C) during the 6-min in vivo electrostimulation protocol and total rate of ATP produced the whole protocol (D). Values are means ± SEM. ^*^Significant difference between GK and WT.

**Fig 5:**
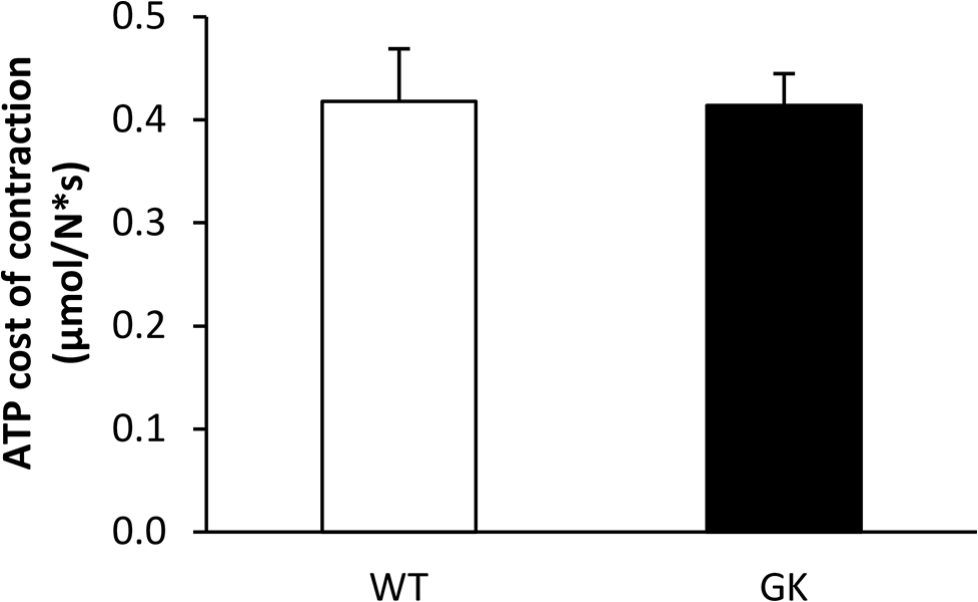
Averaged ATP cost of contraction during the 6-min electrostimulation protocol. Values are means ± SEM.

The time-courses of ATP production rates from CK reaction and oxidative phosphorylation throughout the 6-min ES period did not differ between both groups (Fig 4A and 4B). On the other hand, glycolytic ATP production rate in the early stage of the ES protocol was lower in GK animals (Fig 4C). Overall, the total rate of ATP production (calculated as the sum of ATP produced by the three metabolic pathways over the whole ES protocol) was similar between GK and WT rats (Fig 4D). Besides, the average ATP cost of contraction (calculated across the whole 6-min ES period as the total amount of ATP production scaled to the total force output during the same period) did not differ between GK and Wistar rats (Fig 5).

## Discussion

The causative relationships between insulin-resistance, mitochondrial dysfunction and lipid metabolism alteration are still a matter of debate. The GK model is of interest in order to address this issue given that it is a non-obese T2DM model displaying a normal NEFA plasmatic level and an insulin-resistance [28–32]. Although this model has been widely used for the study of T2DM, data regarding the *in vivo* mitochondrial function are very scarce.

In agreement with previous studies, we found increased plasmatic glucose and decreased insulin level in GK rats whereas both NEFA and IMCL levels were unaltered [30, 46]. These findings are in line with the view that this model exhibits a moderate but stable fasting hyperglycemia early in life and develops beta-cells failure with increasing age, thereby reducing the insulin secretion [29] and leading to insulin resistant state [47, 48].

Our data clearly showed that energy metabolism was disturbed in resting GK muscle. In particular, PCr/ATP ratio and PCr content were both lower, whereas ATP level was unchanged. PCr is considered to play a crucial role in cellular energy metabolism [49]. Actually, PCr level is under the control of creatine kinase (CK), which transfers high-energy phosphate from PCr to ADP to form ATP (via the reaction: PCr + ADP + H^+^ ↔ ATP + creatine) and the PCr-CK system is involved (i) in energy buffering in order to maintain ATP pool charged and (ii) in high-energy phosphate transport between the site of production (mitochondria) and utilization of ATP. Thus, the reduction of PCr content reported herein could indicate that ATP homeostasis was preserved in GK muscle at the expense of PCr stores in order to ensure the basal energy demand. Considering that ATP generation is mainly aerobic at rest [50], these findings might suggest an impaired mitochondrial ATP generation. However, neither of the results we obtained *in vivo* (^31^P-MRS) or *in vitro* (citrate synthase activity assays) support any reduction in mitochondrial capacity, which is consistent with the unaffected mitochondrial respiration reported in isolated mitochondria from GK rat quadriceps muscle [51]. On that basis, the reduced basal PCr content would not be linked to an impaired mitochondrial function. Besides, a decline in basal PCr content has been previously observed as the result of muscle ischemia in association with intracellular acidosis [52]. Interestingly, *in situ* microscopy experiments have reported a disturbed muscle microcirculation in the *spinotrapezius* muscle of GK rats, hence leading to reduced delivery and transport of oxygen [53]. Nevertheless, any disturbed oxygen supply can be dismissed in the present study given that we did not observe a concomitant acidosis in the gastrocnemius muscle but on the contrary a basal alkalosis. On the other hand, considering that ADP stimulates mitochondrial ATP generation through a feedback loop [38] and basal [ADP] was similar in both groups, PCr content reduction we measured in GK muscle could be interpreted as a compensatory mechanism aiming at keeping [ADP] constant in the face of the increased pH_i_ in order to maintain a normal mitochondrial function.

However, the fact that ATP level and mitochondrial function were not altered in resting GK muscle does not necessarily imply that oxidative capacity was preserved in working muscle, i.e., when energy demand may increase substantially. As compared to rest, muscle energy demand can actually increase by several orders of magnitude in exercising muscle in order to maintain ATP homeostasis [54]. In the present study, we implemented an intense fatiguing protocol consisting in 6-min of repeated maximal isometric contractions to produce wide changes in metabolic and mechanical changes. Despite this, we did not measure any alteration of oxidative ATP production in contracting GK muscle. Moreover, the maximal oxidative capacity and the initial rate of post-electrostimulation PCr resynthesis–an *in vivo* index of mitochondrial function [55]–did not differ between both groups thereby indicating that mitochondrial function was not altered in the GK model.

It must be pointed out that the rate of glycolytic ATP production in the early stage of the electrostimulation period was significantly lower in GK muscle. One can assume that this lower rate was linked to the reduced glucose uptake already observed in skeletal muscle of insulin resistant and diabetic patients [56]. This assumption appears however unlikely herein considering the unaltered glycogen content and the even higher glucose content in the GK rats gastrocnemius muscle. The decreased glycolytic flux could rather be related to decreased glucose utilization. In the diabetic state, glucose is indeed preferentially catabolized into the polyol pathway away from energy-producing glycolysis as a result of a reduced glyceraldehydes 3-phosphate dehydrogenase (GAPDH) reaction kinetics and other downstream reactions, e.g., enolase and pyruvate kinase [57].

Another interesting result is that mechanical performance was reduced in GK rats. The lower (-34%) absolute force developed throughout the fatiguing protocol is in line with the reduced muscle strength observed in diabetic patients [4]. Interestingly, this lower force-generating capacity was not fully accounted by the smaller size of the GK gastrocnemius muscle measured from anatomic MR images. Indeed, specific force (i.e., absolute force scaled to muscle size) was also reduced in the diabetic animals, but to a lower extent (-22%), which indicates that additional mechanisms are involved in the impairment of muscle performance. Limitation of energy supply is considered to play a major role in the failure of muscle to sustain force [58], but such an issue is unlikely in contracting GK muscle given that we found that throughout the whole electrostimulation period, ATP level did not fall down to any critical threshold and was even higher than the corresponding level in Wistar rats, hence indicating that the rate of ATP regeneration was not compromised. Further, it can be dismissed that a proportion of ATP produced in contracting GK muscle would be wasted in non-contractile processes because we found that ATP cost of contraction, i.e., the contractile efficiency, was not altered in these animals. Our findings might be at a first glance considered as opposite to that of a previous calorimetric study showing that basal energy expenditure is increased in patients with congenital insulin resistance [59]. Nevertheless, it must be kept in mind that this increased energy expenditure was measured at rest at the whole body level, and might differ from the results we obtained at the skeletal muscle level. Besides, an attractive explanation for the reduced mechanical performance would rather lie in the diabetic neuropathy previously reported in GK model [60]. This nerve disorder causes indeed motor dysfunction leading to muscle weakness and atrophy [61–63]. Diabetic neuropathy could further, in combination to increased autophagy already reported in this model [64], explains the smaller size of gastrocnemius muscle we reported herein.

In conclusion, oxidative ATP generation capacity at rest and during sustained fatiguing electrostimulation protocol was not altered in the GK rat model. These findings provide *in vivo* evidence that insulin resistance is not caused by a primary defect in mitochondrial function in this diabetic model.
